# Descriptive Epidemiology of Prion Disease in Japan: 1999–2012

**DOI:** 10.2188/jea.JE20140022

**Published:** 2015-01-05

**Authors:** Yosikazu Nakamaura, Ryusuke Ae, Ichiro Takumi, Nobuo Sanjo, Tetsuyuki Kitamoto, Masahito Yamada, Hidehiro Mizusawa

**Affiliations:** 1 Department of Public Health, Jichi Medical University, Shimotsuke, Tochigi, Japan; 1 自治医科大学公衆衛生学教室; 2 Department of Neurosurgery, Nippon Medical School Musashi-Kosugi Hospital, Kawasaki, Kanagawa, Japan; 2 日本医科大学武蔵小杉病院脳神経外科学; 3 Department of Neurology and Neurological Science, Graduate School, Tokyo Medical and Dental University, Tokyo, Japan; 3 東京医科歯科大学大学院脳神経病態学; 4 Department of Prion Protein Research, Division of CJD Science and Technology, Tohoku University Graduate School of Medicine, Sendai, Japan; 4 東北大学大学院医学系研究科附属創生応用医学研究センタープリオン病コアセンター病態神経学分野; 5 Department of Neurology and Neurobiology of Aging, Kanazawa University Graduate School of Medical Science, Kanazawa, Japan; 5 金沢大学大学院医薬保健学総合研究科脳医科学専攻脳病態医学講座脳老化・神経病態学

**Keywords:** prion diseases, Creutzfeldt-Jakob syndrome, incidence, mortality, secular trends

## Abstract

**Background:**

Epidemiologic features of prion diseases in Japan, in particular morbidity and mortality, have not been clarified.

**Methods:**

Since 1999, the Research Committee has been conducting surveillance of prion diseases, and the surveillance data were used to assess incident cases of prion diseases. For the observation of fatal cases, vital statistics were used.

**Results:**

Both incidence and mortality rates of prion diseases increased during the 2000s in Japan. However, this increase was observed only in relatively old age groups.

**Conclusions:**

The increased number of patients among old age groups might be due to increased recognition of the diseases. If so, the number of cases should plateau in the near future.

## Abstract (ja)

**背景：:**

日本におけるプリオン病の疫学像、特に罹患や死亡は明らかにされていない。

**方法：:**

1999年より研究班はプリオン病のサーベイランスを実施しており、プリオン病罹患例の観察にサーベイランスのデータを用いた。死亡例の観察には人口動態統計を用いた。

**結果：:**

日本ではプリオン病の罹患率、死亡率共に2000年代の観察期間中に上昇した。しかし、この増加は高齢者群のみで観察された。

**結論：:**

高齢者群での患者数の増加はこの疾患の認識が改善したことによるものかもしれない。そうであれば、この増加傾向は近い将来、横ばい傾向に移行する可能性がある。

## INTRODUCTION

In 1996, when the paper indicating the relationship between bovine spongiform encephalopathy (BSE) and the human variant Creutzfeldt-Jakob disease (CJD) was published,^1^ full-scale epidemiologic research for prion diseases, such as CJD, Gerstmann-Sträusslar-Scheinker disease (GSS), and fatal familial insomnia (FFI), started in Japan.^2^ Since 1999, a nationwide surveillance system for prion diseases has been implemented, and patients with prion diseases have been registered.^3^ The epidemiologic features of prion diseases in Japan are summarized as follows^2^^,^^3^: (1) the annual incidence rate is about 1 case per 1 million population, which is similar to the worldwide standard; (2) the incidence rate is highest among those aged in their 60s and 70s; and (3) CJD associated with cadaveric dura mater transplantation is more prevalent in Japan than in other countries.

Over the past decade, the number of patients with prion diseases has increased in Japan. While the reason for this increase is unclear, epidemiologists must consider that increased recognition of the disease may lead to a subsequent increase in the number of patients. In other words, whether prevalence has truly increased or whether some other factors have made the number merely seem increased should be clarified. Detailed observation of the epidemiologic features of prion diseases might shed light on this apparent increase in prevalence.

We conducted this descriptive epidemiologic research with two purposes: to clarify the recent epidemiologic features of prion diseases in Japan and to obtain some hints about the cause of increasing incidence of the diseases in Japan.

## METHODS

We used two data sets in this study. One was the registry data of prion diseases in Japan obtained through the surveillance system conducted by the Surveillance Committee, which is financially supported by the Ministry of Health, Labour and Welfare of the Japanese government.^3^ The system was first implemented in April 1999. There are several routes to obtain information about the existence of potential patients with prion diseases: a mandatory reporting system from physicians, the public-aid-for-treatment system, and clinical examinations.

First, since 1999, prion diseases have been designated as reportable diseases by the Prevention of Infectious Diseases and Medical Care for Infectious Patients Act (Act No. 114 of 1998). When a physician diagnoses a patient as having a prion disease, he or she must report the fact to a local public health center.

Second, prion diseases are designated as diseases qualifying for public aid. Patients with one of the designated diseases receive treatment from a hospital with public aid, and he or she is not required to pay any fee. The aid is based on a claim made by the patient or his or her family to a public health center, so information on the patient is obtained when making the claim.

Lastly, the Surveillance Committee conducts human prion protaineous gene analyses at Tohoku University and cerebrospinal fluid analyses (14-3-3 protein and total tau protein) at Nagasaki University. Physicians who suspect that a patient has a prion disease or who want to certify the diagnosis of a prion disease may send blood or cerebrospinal fluid to these universities with a patient’s informed consent. The cost is covered by research funds from the national government, and the patient and physician are not responsible for paying any fees.

When the Surveillance Committee obtains information about a potential patient through one of these three routes, one of the committee members, who is a neurologist or a psychiatrist familiar with prion diseases, obtains detailed data about the patient by meeting the patient if possible or using hospital records. Based on the obtained data, the Committee members discuss whether or not the patient has a prion disease, and patients recognized to have a prion disease are registered anonymously.

In the present study, we used only data of registered patients with disease onset from 1999 through 2012. We observed epidemiologic features of prion diseases in Japan calculated standardized morbidity rates by prefecture and secular trends of age-specific incidence rates. As shown in Figure 4, the 2012 data are still incomplete, so patients diagnosed in this year were excluded in observation of time and place.

We also used data on vital statistics in Japan from 1999 through 2012 (http://www.e-stat.go.jp/SG1/estat/GL08020101.do?_toGL08020101_&tstatCode=000001028897&requestSender=dsearch). Since 1999, the statistics presented the numbers of fatal cases with prion diseases (ICD-10th; A81.0 [Creutzfeldt-Jakob disease] + A81.8 [Other atypical virus infections of central nervous system]) by prefecture as an infectious disease as well as the fatal numbers by age and sex. Therefore, we calculated standardized mortality ratios by prefecture in addition to age-specific mortality rates in Japan. For calculating 95% confidence intervals (CIs) of standardized morbidity and mortality ratios, we used the table presented by Schoenberg.^4^

In addition to these analyses, standardized morbidity and mortality ratios—the former of which were based on the surveillance data and the latter of which were based on the vital statistics—were compared with the number of neurologists authorized by the Societas Neurologica Japonoca (http://www.kktcs.co.jp/jsn-senmon/secure/senmon.aspx) per population by prefecture.

The age-specific population for incidence and mortality rates by calendar year was that used in vital statistics, while that for calculation of standardized morbidity and mortality ratios by prefecture was the census population in 2005.

## RESULTS

There were 2026 incident cases of prion diseases (854 males and 1172 females) from 1999 through 2012 and 2334 fatal cases (1035 males and 1299 females) from 1999 through 2012 according to vital statistics in Japan. The average annual incidence rate was 1.09 cases per 1 million population (0.95 for males and 1.22 for females, calculated from 1999 through 2011) and average annual mortality rates were 1.32 per 1 million population (1.20 for males and 1.44 for females).

The results below are described according to the three major characteristic headings of descriptive epidemiology: persons, place, and time.^5^

### Persons

Table 1 shows the characteristics of 2026 incident patients with prion diseases in Japan during the 14 years from 1999 through 2012. The number of patients by age class was largest in the group aged 70–79 years, followed by those aged 60–69 years. Of the 2026 patients with prion diseases, 77% was sporadic CJD patients.

**Table 1.** Demographic characteristics of patients with prion diseases in Japan, 1999–2012

	Whole patients		Sporadic CJD^a^		Variant CJD	CJD with dura mater transplantaion		Familiar prion diseases					Unclassified CJD^c^

								Familiar CJD^b^		GGS		FFI	
Total sample	2026	(100)	1550	(77)	1	83	(4)	298	(15)	84	(4)	4	6

**Sex**													
Male	854	(42)	636	(41)	1	35	(42)	136	(46)	41	(49)	3	2
Female	1172	(58)	914	(59)		48	(58)	162	(54)	43	(51)	1	4
Total	2026	(100)	1550	(100)	1	83	(100)	298	(100)	84	(100)	4	6

**Age at onset (years)**													
10–19	3					2	(2)	1	(0)				
20–29	8	(0)				5	(6)	1	(0)	2	(2)		
30–39	29	(1)	12	(1)		7	(8)	1	(0)	9	(11)		
40–49	69	(3)	40	(3)	1	5	(6)	10	(3)	11	(13)	1	1
50–59	304	(15)	212	(14)		20	(24)	30	(10)	40	(48)	2	
60–69	613	(30)	498	(32)		25	(30)	70	(23)	18	(21)	1	1
70–79	738	(36)	590	(38)		17	(20)	123	(41)	4	(5)		4
80–89	245	(12)	186	(12)		2	(2)	57	(19)				
90–99	14		9					5					
Unknown	3		3										
Total	2026	(100)	1550	(100)	1	83	(100)	298	(100)	84	(100)	4	6

Mean	67.9		68.7			57.9		70.7		53.8		54.5	
SD	11.1		9.8			16.2		11.4		10.7		6.4	
Oldest age	94		94			85		93		75		61	
Youngest age	15		30			15		15		22		46	

CJD, Creutzfeldt-Jakob disease; FFI, fatal familial insomnia; GSS, Gerstmann-Sträusler-Scheinker disease; SD, standard deviation.

Percentages are shown in parentheses. Percentages may not add up to exactly 100% because of rounding.

^a^Including CJD without prion protein gene analyses.

^b^Including patients without prion protein gene variation but with family histories of CJD.

^c^Those whose diagnosis has been confirmed as CJD, but whose type of CJD has not been surveyed.

### Place

**Figure 1.**
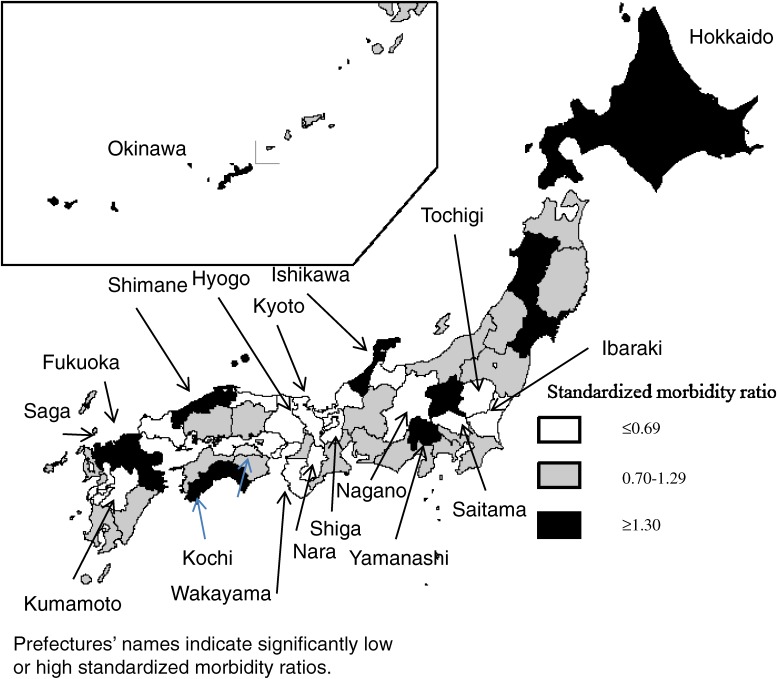
Standardized morbidity ratios of prion diseases in Japan by prefecture, 1999–2011

**Figure 2.**
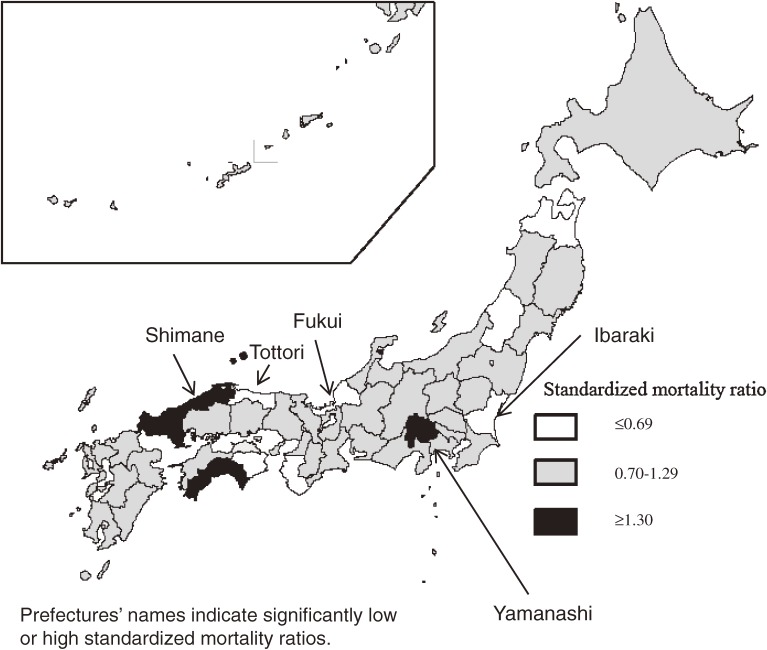
Standardized mortality ratios of prion diseases in Japan by prefecture, 1999–2012

**Figure 3.**
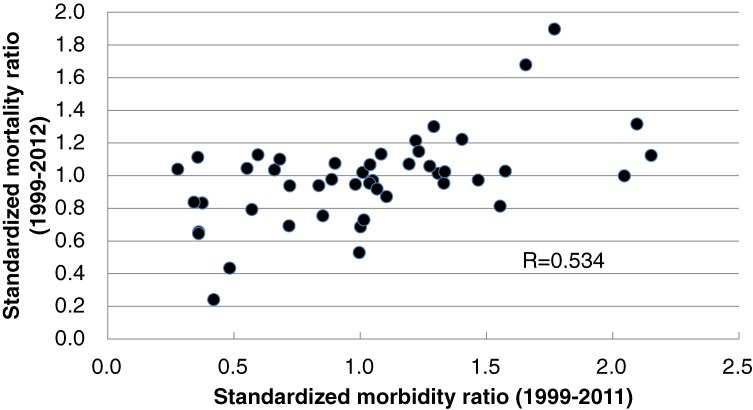
Relationship between standardized morbidity ratios and standardized mortality ratios of prion diseases by prefecture

The standardized morbidity and mortality ratios for each prefecture are shown in Table 2. The standardized morbidity ratios ranged from 0.28 (Shiga Prefecture) to 2.15 (Saga Prefecture), while the ratios for mortality ranged from 0.24 (Tottori Prefecture) to 1.90 (Yamanashi Prefecture). As shown in Figures 1 and 2, no geographical clustering of prevalent prefectures was observed. The correlation coefficient between the morbidity and mortality ratios was 0.53 (95% CI 0.29 to 0.71; Figure 3). We assessed the relationship between the morbidity and mortality of prion diseases and the number of neurologists per population because there is a possibility that shortage of neurological medical services introduces misdiagnosis of prion diseases. However, the correlation coefficient between the standardized morbidity ratio and the number of neurologists was −0.12 (95% CI −0.39 to 0.17), while that for the standardized mortality ratio was −0.09 (95% CI −0.37 to 0.20). The coefficients for the relationship between standardized morbidity and mortality ratios and the number of neurologists per population aged ≥65 years were −0.17 (95% CI: −0.44 to 0.12) and −0.00 (95% CI: −0.29 to 0.29), respectively.

**Table 2.** Morbidity, mortality, and other variables concerning prion diseases in Japan, by prefecture

	Population^a^ (thousands)	Number of newly diagnosed patients	Number of newly diagnosed patients (1999–2011)^b^	Crude incidence rate (per million population/year)	Standardized morbidity ratio (95% CI)	Number of fatal patients (1999–2012)	Crude mortality rate (per million population/year)	Standardized mortality ratio (95% CI)
Total	127 768	2026	1814	1.09		2334	1.30	

Hokkaido	5628	138	124	1.69	1.47 (1.23–1.76)	106	1.35	0.97 (0.80–1.18)
Aomori	1437	19	16	0.86	0.72 (0.41–1.16)	20	0.99	0.69 (0.42–1.07)
Iwate	1385	19	19	1.06	0.85 (0.51–1.35)	22	1.13	0.75 (0.47–1.14)
Miyagi	2360	44	43	1.40	1.31 (0.95–1.77)	43	1.30	1.01 (0.73–1.37)
Akita	1146	28	28	1.88	1.40 (0.93–2.03)	32	2.00	1.22 (0.84–1.72)

Yamagata	1216	20	20	1.26	1.00 (0.61–1.54)	18	1.06	0.69 (0.41–1.09)
Fukushima	2091	38	35	1.29	1.10 (0.77–1.53)	36	1.23	0.87 (0.61–1.20)
Ibaraki	2975	18	15	0.39	0.36 (0.20–0.59)	35	0.84	0.66 (0.46–0.91)
Tochigi	2017	16	10	0.38	0.36 (0.17–0.66)	40	1.42	1.11 (0.79–1.51)
Gunma	2024	41	39	1.48	1.33 (0.95–1.82)	36	1.27	0.95 (0.67–1.32)

Saitama	7054	67	60	0.65	0.66 (0.50–0.85)	119	1.20	1.04 (0.86–1.24)
Chiba	6056	97	85	1.08	1.05 (0.84–1.30)	100	1.18	0.97 (0.79–1.19)
Tokyo	12 577	166	149	0.91	0.90 (0.76–1.06)	228	1.29	1.08 (0.94–1.23)
Kanagawa	8792	154	134	1.17	1.19 (1.00–1.42)	153	1.24	1.07 (0.91–1.26)
Niigata	2431	51	49	1.55	1.28 (0.94–1.68)	53	1.56	1.06 (0.79–1.39)

Toyama	1112	12	10	0.69	0.57 (0.27–1.05)	18	1.16	0.79 (0.47–1.25)
Ishikawa	1174	38	35	2.29	2.05 (1.43–2.84)	22	1.34	1.00 (0.63–1.51)
Fukui	822	7	6	0.56	0.48 (0.18–1.05)	7	0.61	0.43 (0.17–0.89)
Yamanashi	885	26	23	2.00	1.77 (1.12–2.65)	32	2.58	1.90 (1.30–2.68)
Nagano	2196	21	19	0.67	0.55 (0.33–0.86)	47	1.53	1.04 (0.77–1.39)

Gifu	2107	38	32	1.17	1.04 (0.71–1.46)	38	1.29	0.95 (0.68–1.31)
Shizuoka	3792	71	67	1.36	1.22 (0.95–1.55)	86	1.62	1.21 (0.97–1.51)
Aichi	7255	104	94	1.00	1.01 (0.82–1.23)	121	1.19	1.02 (0.85–1.22)
Mie	1867	35	34	1.40	1.23 (0.85–1.72)	41	1.57	1.15 (0.82–1.56)
Shiga	1380	6	5	0.28	0.28 (0.09–0.65)	24	1.24	1.04 (0.67–1.55)

Kyoto	2648	16	14	0.41	0.37 (0.20–0.63)	40	1.08	0.83 (0.59–1.13)
Osaka	8817	109	101	0.88	0.84 (0.68–1.02)	145	1.17	0.94 (0.80–1.11)
Hyogo	5591	61	54	0.74	0.68 (0.51–0.89)	112	1.43	1.10 (0.91–1.33)
Nara	1421	8	7	0.38	0.34 (0.14–0.70)	22	1.11	0.84 (0.53–1.27)
Wakayama	1036	6	6	0.45	0.36 (0.13–0.78)	14	0.97	0.65 (0.35–1.09)

Tottori	607	6	4	0.51	0.42 (0.11–1.07)	3	0.35	0.24 (0.05–0.70)
Shimane	742	23	21	2.18	1.66 (1.02–2.53)	28	2.69	1.68 (1.12–2.43)
Okayama	1957	32	30	1.18	1.01 (0.68–1.45)	28	1.02	0.73 (0.49–1.06)
Hiroshima	2877	46	41	1.10	0.98 (0.70–1.33)	51	1.27	0.95 (0.71–1.24)
Yamaguchi	1493	35	32	1.65	1.29 (0.88–1.82)	42	2.01	1.30 (0.94–1.76)

Tokushima	810	16	13	1.23	1.00 (0.53–1.70)	9	0.79	0.53 (0.26–1.01)
Kagawa	1012	16	14	1.06	0.89 (0.48–1.49)	20	1.41	0.98 (0.60–1.51)
Ehime	1468	26	25	1.31	1.07 (0.69–1.58)	28	1.36	0.92 (0.61–1.33)
Kochi	796	28	28	2.70	2.10 (1.39–3.04)	23	2.06	1.32 (0.83–1.97)
Fukuoka	5050	120	111	1.69	1.57 (1.30–1.90)	93	1.32	1.03 (0.83–1.26)

Saga	866	29	28	2.49	2.15 (1.43–3.12)	19	1.57	1.12 (0.68–1.75)
Nagasaki	1479	27	25	1.30	1.08 (0.70–1.60)	34	1.64	1.13 (0.78–1.59)
Kumamoto	1842	20	17	0.71	0.60 (0.35–0.95)	42	1.63	1.13 (0.81–1.52)
Oita	1210	27	26	1.65	1.34 (0.87–1.96)	26	1.54	1.02 (0.67–1.51)
Miyazaki	1153	14	13	0.87	0.72 (0.38–1.23)	22	1.36	0.94 (0.59–1.42)

Kagoshima	1753	32	29	1.27	1.04 (0.70–1.50)	39	1.59	1.07 (0.76–1.46)
Okinawa	1362	25	24	1.36	1.55 (1.00–2.32)	16	0.84	0.81 (0.47–1.32)

Unknown		30				1		

CI, confidence interval.

^a^Data from 2005 Census.

^b^Patients whose address was unknown were excluded.

### Time

**Figure 4.**
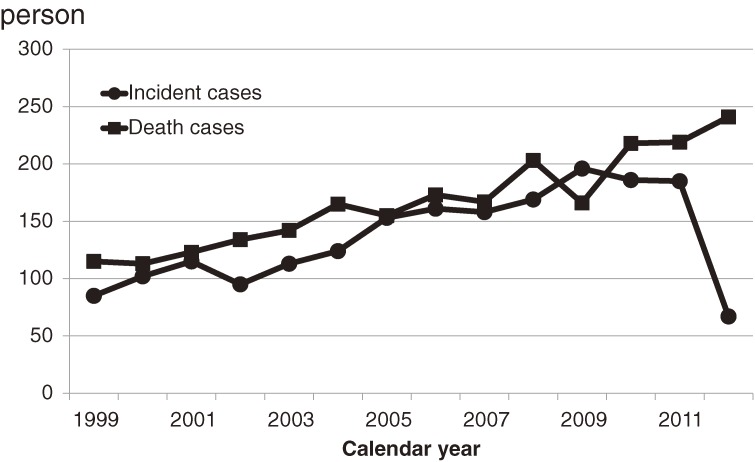
Numbers of incident cases of and deaths from prion diseases in Japan, 1999–2012

**Figure 5.**
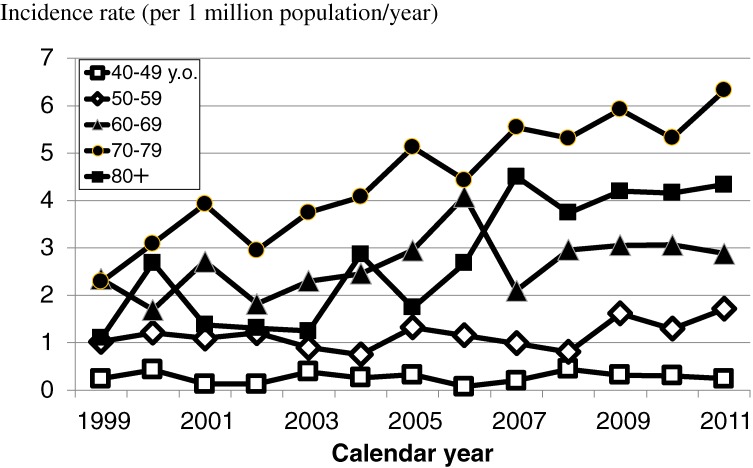
Annual incidence rates of prion diseases in Japan by age

**Figure 6.**
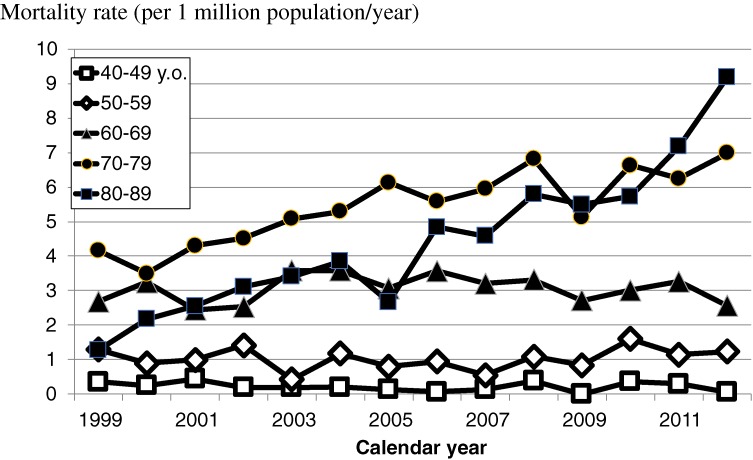
Annual mortality rates of prion diseases in Japan by age

Figure 4 shows the annual incidence and mortality cases by year. Because the cases of prion diseases in recent years have not all been discussed by the Committee yet, the numbers of new cases in recent years (specifically 2012) were small. Figures 5 and 6 show the age-specific incidence and mortality rates of prion diseases by year. Although the rates increased among older subjects, those among younger subjects did not increase.

## DISCUSSION

In the present study, we described the epidemiologic features of prion diseases in Japan using two data sources—the prion disease surveillance system and vital statistics—from three descriptive epidemiologic viewpoints: persons, place, and time.

The prion disease surveillance system in Japan intends to collect information when a person receives a prion disease. The vital statistics comprise the data when the patient dies. According to the natural history of the disease, which is that patients with prion diseases die within a few years of disease onset, the numbers of incident patients and deceased ones should be similar, although they were not identical because of the time lag between onset and death. We recognized 2026 incident cases and 2334 fatal cases in this study for the observed 14 years. There are several possible explanations for the difference between the two figures (308 cases). First, as mentioned before, a time lag exists between onset and death. Second, the surveillance data were incomplete for recent calendar years (Figure 4). In addition, discrepancies in diagnoses may exist between the two databases. Cases in the surveillance system should be true prion diseases because their diagnoses were based on the discussion of the Surveillance Committee, which consists of neurologists, psychiatrists, and neuropathologists and uses clinical findings and medical records gathered by the committee members, gene analyses, and pathological findings including results of western blot analyses. On the other hand, the vital statistic data consists of death certificate data for which the recorded cause underlying death was a prion disease. Because all physicians are able to create death certificates, some of these might be cases described by physicians who were not experts in prion diseases. These points might be the reasons for the gap between the numbers of incident and fatal cases. Nonetheless, we consider the validity of the two datasets used in this study to be quite high and believe that our findings accurately reflect the true epidemiologic features of prion diseases in Japan.

Different epidemiologic aspects from those of prion diseases in European countries and North America^6^^,^^7^ were observed in Japan in the present study. The proportion of patients with acquired CJD among all prion disease patients was high. Many of them developed CJD following cadaveric dura mater transplantation.^8^^–^^12^ Currently, we have data of 147 such cases, and detailed epidemiologic features will be presented in another article. On the other hand, we observed only a single case of acquired prion diseases other than dura-related CJD, which was a case of variant CJD in 2005.^13^^,^^14^

Although there was no geographical clustering of prefectures with high incidence rates or mortality rates, some prefectures presented high rates. For example, both incidence and mortality rates were high in Yamanashi prefecture. There have been several articles about familial clustering of prion diseases in this prefecture.^15^^–^^19^ Of the 23 patients reported in Yamanashi, 57% (13 cases) had familial CJD, although only 15% of prion disease patients had familial CJD in Japan as a whole, as shown in Table 1.

The numbers of incident and fatal patients increased in Japan during the last decade, as shown in Figure 4, although no chronological changes in the number of patients with prion diseases were observed in European countries.^20^ The increase in number of fatal cases was reflected in the increase in number of incident cases in our study. As shown in Figures 5 and 6, the increases in numbers of incident and fatal cases were also reflected in the increased number of cases among the elderly. This phenomenon might be due to the substantial network of gene and spinal fluid analytic systems in Japan.

The Surveillance Committee has publicized the system not only to neurologists but also to general physicians. A patient with rapidly progressive dementia dying before diagnosis might be diagnosed with a prion disease through these gene and/or cerebrospinal fluid analyses. If a physician uses these systems, the Surveillance Committee is automatically notified of the existence of a potential patient, starts getting information about the patient, and discusses whether or not the patient has a prion disease. Further dissemination of information about the analytic system might increase the rate of identification of such patients, particularly among older patients.

The number of gene analyses conducted at Tohoku University increased from 132 cases in 1999 to 273 in 2012. Given that relatively young patients (ie, in their 40s or 50s) with rapidly progressing dementia are rare, such patients are typically referred to specialists in dementia (including prion diseases), so any issues with recognition have been negligible. If knowledge of the gene and spinal fluid analyses system is propagated to all physicians in this country, the issue of recognition should be diminished even further, and the chronological increase of the incidence rate should plateau. However, despite this expected plateau in the near future, the number of patients may still increase because of the growing number of old people in Japan.

Selection bias and information bias may have affected this study. The Surveillance Committee has made an effort to obtain information for all patients with prion diseases in Japan, but the database is not complete. Similar selection bias may be present in the vital statistics data as well; those with prion diseases whose deaths were attributed to conditions other than prion diseases would not be counted as prion disease deaths. Information bias may also exist on the vital statistics data; we were unable to clarify the validity of the diagnosis on death certificates, whereas diagnoses obtained through the surveillance system were validated by the Committee members, including neurologists and neuropathologists.

In conclusion, we showed here the epidemiologic features of prion diseases in Japan. Increased recognition of prion diseases may account for the observation of chronological increases in the number of patients with prion diseases, and the increasing trend of numbers of patients might soon plateau.

## ONLINE ONLY MATERIAL

Abstract in Japanese.
