# Supplementary material for: Descriptive Epidemiology of Prion Disease in Japan: 1999–2012
Source: J Epidemiol. 2015 Jan 5;25(1):8–14. doi: 10.2188/jea.JE20140022 (PMC4275432; doi:10.2188/jea.JE20140022)
Supplement: Abstract in Japanese. [file je-25-008-s001.pdf]

日本におけるプリオン病の記述疫学：1999～2012 年

中村好一[1]、阿江竜介[1]、太組一朗[2]、三條伸夫[3]、北本哲之[4]、山田正仁[5]，水澤英洋[3]

[1] 自治医科大学公衆衛生学教室、[2] 日本医科大学武蔵小杉病院脳神経外科学、[3] 東京医科歯科大学大学院脳神経病態学、[4] 東北大学大学院医学系研究科附属創生応用医学研究センタープリオン病コアセンター病態神経学分野、[5] 金沢大学大学院医薬保健学総合研究科脳医科学専攻脳病態医学講座脳老化・神経病態学

背景：日本におけるプリオン病の疫学像、特に罹患や死亡は明らかにされていない。

方法：1999 年より研究班はプリオン病のサーベイランスを実施しており、プリオン病罹患例の観察にサーベイランスのデータを用いた。死亡例の観察には人口動態統計を用いた。

結果：日本ではプリオン病の罹患率、死亡率共に 2000 年代の観察期間中に上昇した。しかし、この増加は高齢者群のみで観察された。

結論：高齢者群での患者数の増加はこの疾患の認識が改善したことによるものかもしれない。そうであれば、この増加傾向は近い将来、横ばい傾向に移行する可能性がある。

キーワード：プリオン病、クロイツフェルト・ヤコブ病、罹患、死亡、時系列変化
